# A rare germline *CDKN2A* variant (47T>G; p16-L16R) predisposes carriers to pancreatic cancer by reducing cell cycle inhibition

**DOI:** 10.1016/j.jbc.2021.100634

**Published:** 2021-04-03

**Authors:** Isaac P. Horn, David L. Marks, Amanda N. Koenig, Tara L. Hogenson, Luciana L. Almada, Lauren E. Goldstein, Paola A. Romecin Duran, Renzo Vera, Anne M. Vrabel, Gaofeng Cui, Kari G. Rabe, William R. Bamlet, Georges Mer, Hugues Sicotte, Cheng Zhang, Hu Li, Gloria M. Petersen, Martin E. Fernandez-Zapico

**Affiliations:** 1 Division of Oncology Research, Schulze Center for Novel Therapeutics, Mayo Clinic, Rochester, Minnesota, USA; 2 Division of Biochemistry and Molecular Biology, Mayo Clinic, Rochester, Minnesota, USA; 3 Division of Biomedical Statistics and Informatics, Mayo Clinic, Rochester, Minnesota, USA; 4 Division of Molecular Pharmacology and Experimental Therapeutics, Mayo Clinic, Rochester, Minnesota, USA; 5 Division of Epidemiology, Mayo Clinic, Rochester, Minnesota, USA

**Keywords:** cell proliferation, cell cycle, CDK4, cancer biology, genetic disease, inherited mutation, melanoma, fibroblast, pancreatic adenocarcinoma, BB, blocking buffer, CDK, cyclin-dependent kinase, HSF, human skin fibroblast, MD, molecular dynamics, PBS-T, PBS, 0.3% Tween 20, PDAC, pancreatic adenocarcinoma, SE, standard error

## Abstract

Germline mutations in *CDKN2A*, encoding the tumor suppressor p16, are responsible for a large proportion of familial melanoma cases and also increase risk of pancreatic cancer. We identified four families through pancreatic cancer probands that were affected by both cancers. These families bore a germline missense variant of *CDKN2A* (47T>G), encoding a p16-L16R mutant protein associated with high cancer occurrence. Here, we investigated the biological significance of this variant. When transfected into p16-null pancreatic cancer cells, p16-L16R was expressed at lower levels than wild-type (WT) p16. In addition, p16-L16R was unable to bind CDK4 or CDK6 compared with WT p16, as shown by coimmunoprecipitation assays and also was impaired in its ability to inhibit the cell cycle, as demonstrated by flow cytometry analyses. *In silico* molecular modeling predicted that the L16R mutation prevents normal protein folding, consistent with the observed reduction in expression/stability and diminished function of this mutant protein. We isolated normal dermal fibroblasts from members of the families expressing WT or L16R proteins to investigate the impact of endogenous p16-L16R mutant protein on cell growth. In culture, p16-L16R fibroblasts grew at a faster rate, and most survived until later passages than p16-WT fibroblasts. Further, western blotting demonstrated that p16 protein was detected at lower levels in p16-L16R than in p16-WT fibroblasts. Together, these results suggest that the presence of a *CDKN2A* (47T>G) mutant allele contributes to an increased risk of pancreatic cancer as a result of reduced p16 protein levels and diminished p16 tumor suppressor function.

Germline mutations in *CDKN2A* affecting the encoded p16^INK4A^ protein (hereafter referred to as p16) are a major risk factor for familial melanoma, with a high proportion (40–60%) of families in which multiple individuals develop melanoma-carrying germline mutations in *CDKN2A* (1). In addition, germline *CDKN2A* mutations are associated with higher risk of pancreatic adenocarcinoma (PDAC), with some evidence for increased cancers at other sites (2, 3, 4, 5, 6, 7). Somatic alterations and silencing of p16 occur in almost all PDAC tumors and frequently occur in melanoma as well (8, 9, 10, 11). Thus, p16 inactivation plays an important role in the development of both pancreatic cancer and melanoma. p16 is a known tumor suppressor that functions by binding to and inhibiting cyclin-dependent kinase (CDK) 4 and CDK6, preventing the formation of cyclin D/CDK4/6 complexes, thereby inhibiting retinoblastoma (RB1)-dependent cell proliferation (12). It is assumed that mutations in p16 could lead to decreased cell cycle regulatory properties of this protein. A number of p16 mutations have been demonstrated to be epidemiologically linked to melanoma and/or determined to have reduced functionality using *in vitro* assays (1, 13, 14, 15). However, many p16 variants of unknown significance have been identified in multiple melanoma families.

Our group identified four familial melanoma/pancreatic cancer kindreds segregating a rare germline missense variant, *CDKN2A* (47T>G), that encodes the variant protein p16-L16R. Although CDKN2A encodes two distinct tumor suppressor proteins by alternative splicing, p16, and p14ARF, the 47T>G nucleotide alteration only affects the sequence of p16. The L16 residue of p16 is evolutionarily conserved throughout eutherian mammals, marsupials, and birds (4). Previous *in silico* analysis suggests that the p16-L16R variant may be pathogenic (16); however, its functionality has not been directly tested. We investigated the functional significance of this variant using a combination of biochemical, molecular, and cell biological techniques to determine the mechanisms by which expression of p16-L16R might lead to increased carcinogenesis. Ectopic expression studies in p16-null pancreatic cancer cells demonstrated that p16-L16R exhibited lower protein levels than wild-type (WT) p16 (p16-WT) and that p16-L16R almost completely lacked cell cycle inhibitory function. Studies that compared primary normal skin fibroblasts derived from p16 WT with L16R individuals from these melanoma/pancreatic cancer kindreds showed that the p16-L16R fibroblasts had increased growth rates compared with WT fibroblasts, especially among p16-L16R cells obtained from donors who had been diagnosed with multiple cancers. These studies suggest that possession of the L16R-p16 variant leads to a deficiency in cell cycle regulation, predisposing these individuals to higher risk of developing cancer.

## Results

### *CDKN2A* (47T>G)/p16-L16R is a novel variant found in several familial pancreatic cancer and melanoma kindreds

Members of four multigeneration pedigrees ascertained through pancreatic cancer probands were recruited by genetic epidemiologists at Mayo Clinic. Among an aggregate total of 127 individuals recruited, there were nine individuals who had pancreatic cancer, 23 with malignant melanoma, and three with both cancers. Probands in these pedigrees carried the germline variant, *CDKN2A* (47T>G)/p16-L16R, confirmed by a commercial genetic testing laboratory (16, 17). Further germline DNA sequencing was performed on available samples of 70 subjects aged 30 and older who were members of three kindreds in which a proband with pancreatic cancer carried the *CDKN2A* (47T>G) mutation. There were 30 individuals who were found not to carry the mutation (age range 30–71); among these noncarriers there were five reported cancers (colorectal cancer, breast cancer, cervical cancer, renal cancer, and pancreatic neuroendocrine tumor); none reported having multiple cancers. The remaining noncarriers reported no cancer history. In contrast, among 40 kindred members who were mutation carriers (age range 31–73), more cancers were reported: two carriers had pancreatic cancer only, one carrier had pancreatic cancer and melanoma, and one carrier had pancreatic cancer and colorectal cancer. Thirteen carriers had melanoma only, and three carriers reported other cancers (breast, colorectal cancer). Among these carriers with cancer, four reported having additional cancers. The segregation of the mutation in these kindreds is consistent with autosomal dominant inheritance of the cancer phenotypes. These findings demonstrate a higher occurrence of cancer in 16-L16R carriers (50%) than noncarriers (16.7%) within these kindreds. Similarly, a kindred with the p16-L16R mutation was previously reported in which six out ten carriers presented with melanoma (18).

### Structural modeling of p16-L16R revealed altered conformations that may disrupt protein–protein interactions

**Figure 1:**
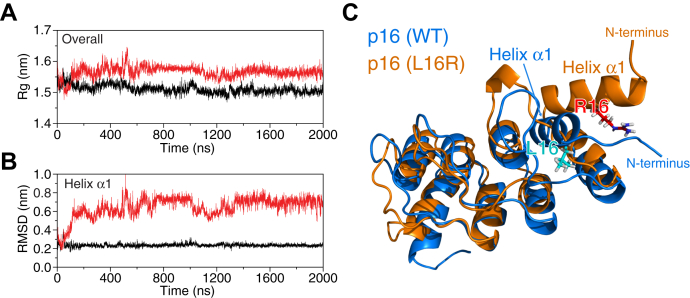
**Molecular dynamics simulations predict altered conformation of p16-L16R.** The structures of WT and L16R p16 were predicted based on the nuclear magnetic resonance (NMR) solution structure of WT p16 (20). Time evolution of radius of gyration (*A*) and backbone root-mean-square deviation of atomic position (RMSD) (*B*) with respect to the starting structures during MD simulations. The *black lines* represent p16-WT and the *red lines* indicate p16-L16R. The radius of gyration values were calculated for p16 (residues 8–139) while the backbone RMSD values were calculated for p16 helix α1 (residues 12–22). *C*, structural overlay of p16 wild type (WT, *blue*) and L16R mutant (*orange*) both at 800 ns of simulation. The side chains of Leu16 and Arg16 are in *stick* representation.

To evaluate the effect of the L16R mutation on the structure of p16, we ran 2 μs molecular dynamics (MD) simulations of WT and L16R mutant using the nuclear magnetic resonance (NMR) spectroscopy solution structure of p16 as a starting model (19). While the WT system remained stable in the course of the simulation, there was a clear change in conformation early in the simulation for the p16-L16R mutant (Fig. 1, *A* and *B*). This conformational change is not surprising as Leu16 is buried in WT-p16. Replacement of Leu16 by a bulky, positively charged residue (Arg16) would therefore be expected to disrupt the protein structure. Indeed, we observed clear opening of p16 helix α1 that harbors Arg16 after 100 ns of simulation (Fig. 1*C*). This dramatic change in conformation most likely disrupts the interaction of p16 with its binding partners. We note that helix α1 contributes to intermolecular contacts in the X-ray structure of p16 in complex with CDK4 (20). Furthermore, since helix α1 is important for the integrity of the p16 fold, another likely outcome of the L16R mutation is the global aggregation of p16.

### p16-L16R exhibits diminished cell cycle inhibition and reduced protein levels

**Figure 2:**
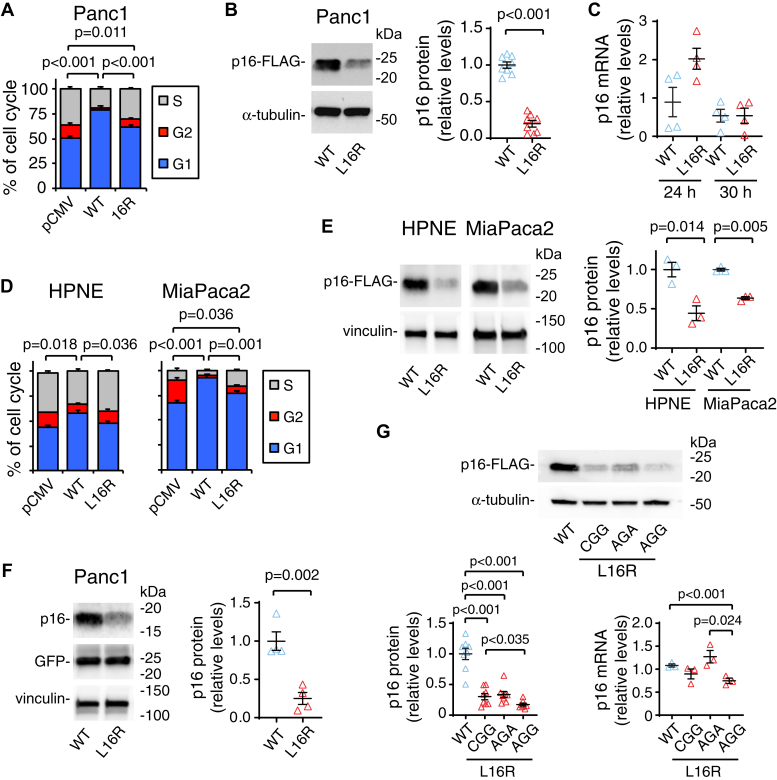
**p16-L16R exhibits diminished ability to inhibit cell cycle progression and reduced protein levels.** FLAG-tagged p16 WT, L16R, or pCMV control vector, plus a vector encoding GFP were transiently transfected into Panc1 (*A*), MiaPaca2, and HPNE cells (*D*) using equal μg of plasmid DNA. Forty-eight hours after transfection, cell cycle analysis of transfected cells was performed using FACS. Results are means ± SE and are expressed as percentages of the population in each cell cycle phase. n = 7 for each group for Panc1, n = 4 for MiaPaca2, and n = 3 for HPNE cells. *Brackets* indicate significant differences between the sum of G2 + S values between groups. After 48 h of transfection with p16 WT or L16R, lysates from transfected Panc1 cells (*B*), MiaPaca2, and HPNE cells (*E*) were analyzed by western blotting. *Left panels*, western blot showing reduced L16R *versus* WT p16 protein levels. α-Tubulin or vinculin was blotted as a protein loading control. *Right panel*, quantitation of p16 protein expression from western blots. Results are from eight (Panc1) or four (MiaPaca2 and HPNE) independent experiments and are shown as individual replicates (*triangle markers*) and mean ± SE (*black crossbars*) normalized to the level for WT expression. *C*, after transfection of Panc1 cells with WT and L16R p16, RNA was extracted at 24 and 30 h. p16 mRNA levels were quantitated by qPCR. Values are mean ± SE and are expressed as relative values normalized to housekeeping gene expression. n= 3 for each group. *E*, Panc1 cells were transfected for 48 h with untagged p16 WT and L16R in the pIRES2-EGFP bicistronic vector. *Left panel*, western blot showing reduced L16R *versus* WT p16 protein levels. GFP and vinculin were blotted as transfection/protein loading controls. *Right panel*, quantitation of p16 protein expression from western blots. Results are from four independent experiments and are expressed as mean ± SE normalized to the level for WT expression. *F*, cells were transfected for 40 h with vectors encoding p16-WT, p16-L16R using the arginine codon (CGG) found at amino acid 16 in the L16R variant, or two alternate codons for arginine (AGA and AGG) at this site. *Upper panel*, a representative western blot showing expression of each p16 form; *lower left panel*, quantitation of p16 protein expression. n = 8 for each construct. Results are expressed as mean ± SE normalized to the level for WT expression. *Lower right panel* shows relative mRNA expression levels for each p16 construct 40 h after transfection. Results are means ± SE expressed as in *panel C*. n= 3 for each group. For all graphs, *brackets* with *p* values above indicate groups that are significantly different in two-tailed *t*-tests. Pairs of groups *without brackets* are not significantly different.

To assess the functional consequences of the L16R mutation, p16-null Panc1 and MiaPaca2 pancreatic cancer cells (21), and an hTERT-immortalized human pancreatic epithelial nestin-expressing cell line that expresses KRAS^G12V^ and an shRNA against p16 (22) (referred to HPNE cells hereafter) were transiently transfected with FLAG-tagged p16-WT or p16-L16R. After 48 h, the effects of the p16 isoforms on the cell cycle were measured by flow cytometry. Transfection with p16-WT significantly decreased cell division in all three cell lines as shown by a decrease in the percentage of cells in the sum of G2 and S phases compared with transfection with the pCMV control vector (Fig. 2, *A* and *D*). In contrast, transfection with p16-L16R elicited only a small decrease in the proportion of cells in the sum of G2 and S phases compared with controls, and the decrease in G2+S was significantly less than that observed with p16-WT (Fig. 2, *A* and *D*). The transfection efficiency for p16 in Panc1 cells was shown to be ∼17% for WT and 18% for L16R p16 as estimated by immunofluorescent staining (Fig. S1), suggesting that differences in WT *versus* L16R p16 could not be explained by differential transfection rates. Western blotting using an anti-FLAG antibody after 48 h of transfection with FLAG-tagged p16-WT or p16-L16R demonstrated significantly lower levels of p16-L16R compared with p16-WT on all three cell lines (Fig. 2, *B* and *E*). These data indicate that the p16-L16R variant has reduced protein levels and is deficient in suppressing G1-S phase progression compared with the p16-WT under the same transfection conditions.

p16-FLAG mRNA expression was not significantly different between WT and mutant p16 transfections in Panc1 cells at 24 and 30 h (Fig. 2*C*), indicating that decreased p16-L16R protein levels cannot be explained by reduced p16-L16R mRNA expression. Transfection of untagged WT and L16R p16 in Panc1 cells and detection using a p16 antibody also showed significantly lower levels of L16R than WT p16 (Fig. 2*F*), indicating that reduced expression of L16R is not influenced by the presence of the FLAG epitope tag. Since individual codons can have an impact on protein translational efficiency and protein folding (23, 24), we considered the possibility that the alteration of codon 16 of p16 from CUG in p16-WT mRNA to CGG in the L16R variant mRNA might influence the efficiency of translation of the p16 protein. Thus, L16R constructs were designed with alternate arginine codons AGA and AGG at this position, expressed by transfection, and tested for protein expression. However, each of these alternative codons resulted in decreased protein levels compared with p16-WT (Fig. 2*G*). p16-L16R with codons CGG and AGA exhibited mRNA levels that were not significantly lower than that of WT p16 (Fig. 2*E*). AGG-L16R p16 had significantly lower mRNA levels than p16-WT and AGA-L16R p16, suggesting that there might be a codon bias effect against the AGG codon. (Fig. 2*G*, lower right panel). Together, these data suggest that the decreased protein level observed for p16-L16R is likely determined by a posttranscriptional mechanism.

**Figure 3:**
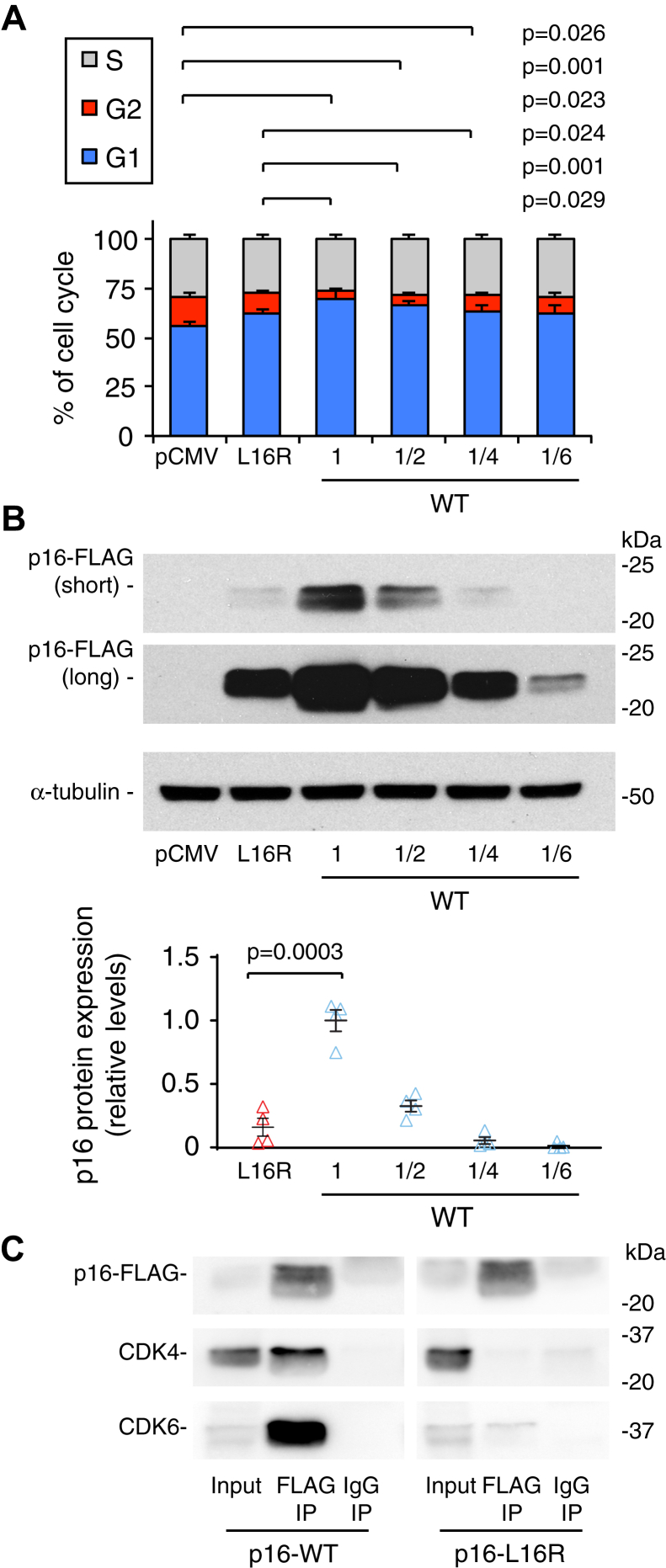
**p16-L16R protein is deficient in cell cycle suppression activity and interaction with CDK4/6.***A* and *B*, Panc1 cells were transfected with pCMV, p16-L16R, or different ratios of p16-WT DNA (1/6, 1/4, 1/2, or 1) relative to L16R DNA. For p16-WT transfections, pCMV DNA was added to make total DNA amounts (5 μg/10 cm dish), the same per transfection. Cells were cotransfected with GFP to label transfected cells. After 1 day, cells were split into two replicate dishes for each condition. *A*, after another 24 h in culture, cells were sorted by FACS to measure the proportions of cell populations in different phases of the cell cycle. Values are means ± SE and are expressed as percentages of cells in each cell cycle phase. n = 4 for each group. *Brackets above bars* with *p* values to the *right* indicate groups that are significantly different (2-tailed T-tests) in G2 phase level. *B*, replicate dishes were lysed after 2 days transfection and analyzed by western blotting. *Upper panel*, western blot showing expression of WT and L16R. Short and long exposures of the p16-FLAG blot are shown. α-Tubulin blotting is also shown. *Lower panel*, quantitation of protein levels from p16 blots. n = 4 for each group. Results are individual replicates (*triangle markers*) and means ± SE expressed relative to the value for p16-WT transfected at equal DNA levels as p16-L16R. *Brackets* with *p* values indicate groups that are significantly different in two-tailed *t*-tests. *C*, Panc1 cells were transfected with FLAG-tagged WT or L16R p16. The WT form was transfected using 1/3 the DNA relative to the L16R construct to yield similar levels of protein expression for both variants. After 48 h, lysates were immunoprecipitated with anti-FLAG or with nonspecific IgG. Results were analyzed by western blotting for FLAG-p16, CDK4, and CDK6. Note that CDK4 and CDK6 strongly co-immunoprecipitated with p16-WT but not with p16-L16R.

The functionality of the L16R mutant protein in cell cycle suppression was further evaluated by transfecting p16-L16R *versus* different amounts of p16-WT DNA and performing flow cytometry 48 h after transfection. The L16R mutant exhibited significantly less cell cycle suppression than the WT transfected at DNA ratios of 1, ½, or 1/4 compared with p16-L16R and was similar in cell cycle suppression effects to the WT expressed using 1/6 of the DNA compared with p16-L16R (Fig. 3*A*). However, western blotting of cells from the same transfection experiments showed that p16-L16R protein expression was higher than that of the 1/4 ratio p16-WT transfection (Fig. 3*B*). These results indicate that the L16R mutant is less functional than the WT p16 even when protein levels are equivalent.

The L16R mutant was evaluated by its ability to coimmunoprecipitate endogenous CDK4 and CDK6. Panc1 cells were transfected with p16-L16R or with a reduced DNA amount of p16-WT to yield a similar level of protein. After 48 h, cell lysates were immunoprecipitated using an anti-FLAG antibody and subsequently analyzed by western blotting for FLAG-p16, CDK4, and CDK6. The L16R mutant showed almost no co-immunoprecipitation with CDK4/6, whereas the p16-WT co-immunoprecipitated appreciable levels of CDK4 and CDK6 (Fig. 3*C*), indicating that the p16-L16R has a reduced affinity for CDK4/6. These results are consistent with the diminished ability of p16-L16R to inhibit cell cycle progression (Figs. 2*A* and 3*A*).

To gain further insight into the mechanisms underlying the increased stability of WT *versus* L16R p16, p16-WT protein expression was tested after the knockdown of CDK4 or CDK6 to determine if WT-p16 stability is affected by loss of binding to CDK4/6. Cells were treated for 24 h with siRNA against CDK4, CDK6, or a nontargeting control. Cells were then transfected with p16-WT for 48 h and analyzed by western blotting. Although CDK4 and CDK6 protein levels were decreased ∼90% by their respective siRNAs, there was no change in WT-p16 expression with knockdown of either CDK4 or CDK6 (Fig. S2). Thus, the binding of CDK4 or CDK6 to p16 apparently has little impact on p16 stability or targeting for degradation and suggests that p16-L16R instability in not related to its inability to bind CDK4 or CDK6. We next investigated whether proteasomal degradation played a role in the differential levels of WT *versus* L16R p16. In Panc1 cells, we found that overexpressed p16-L16R-FLAG (Fig. S3*A*) and untagged p16-L16R (Fig. S3*B*) protein levels were increased by treatment with the proteasome inhibitor MG132, but did not reach p16-WT levels. p16-L16R-FLAG protein levels were increased up to p16-WT-FLAG levels upon treatment with another proteasome inhibitor, bortezomib, when expressed in Panc1 cells and RPM1-7951 melanoma cells, a cell line homozygous for p16-L16R (Fig. S3, *C* and *D*). These results suggest that ectopically expressed p16-L16R protein is more rapidly degraded by the proteasome that the WT protein and is stabilized by bortezomib.

### Characterization of other CDKN2A variants associated with PDAC and melanoma

In previous studies, we identified a large number of novel CDKN2A variants of unknown significance in addition to p16-L16R that were associated with an increased risk of PDAC and melanoma (2, 25). We compared a series of these variants to WT and L16R p16 for protein expression and cell cycle functionality using the methods described above. Several of these variants had lower protein expression compared with p16-WT (Fig. S4*A*). However, only the L16R mutant and one variant (Δ86–92) containing a seven amino acid deletion showed reduced cell cycle suppression compared with the WT protein (Fig. S4*B*). Thus, under our experimental conditions, we were unable to identify any loss of function for most of these variants which might explain their association with cancer risk. It is possible that differences in function of these variants were not ascertainable using assays based on p16 overexpression, or that these variants affect other noncanonical functions of p16 (*e.g.*, p53 stabilization, regulation of reactive oxygen species), which we have not assessed (26, 27, 28).

### Increased proliferation rates in skin fibroblasts derived from p16-L16R family members

The above studies demonstrate that p16-L16R has reduced protein expression and diminished ability to suppress cell cycling compared with p16-WT when expressed ectopically in p16-null Panc1 cells. We next wanted to determine if any loss of p16-L16R function could be detected when this protein is expressed endogenously, as occurs in human carriers. Thus, normal human skin fibroblasts (HSFs) were isolated from punch biopsies taken from p16-L16R family members with L16R (n = 8) and WT (n = 7) p16 genotypes and used as a surrogate for cells that might be at risk of transformation as a result of p16-L16R carriage. Genomic DNA sequencing verified that all HSFs from previously identified L16R individuals were heterozygous for *CDKN2A* (47T>G)/p16-L16R allele, and HSFs from those identified as noncarriers were p16-WT.

**Figure 4:**
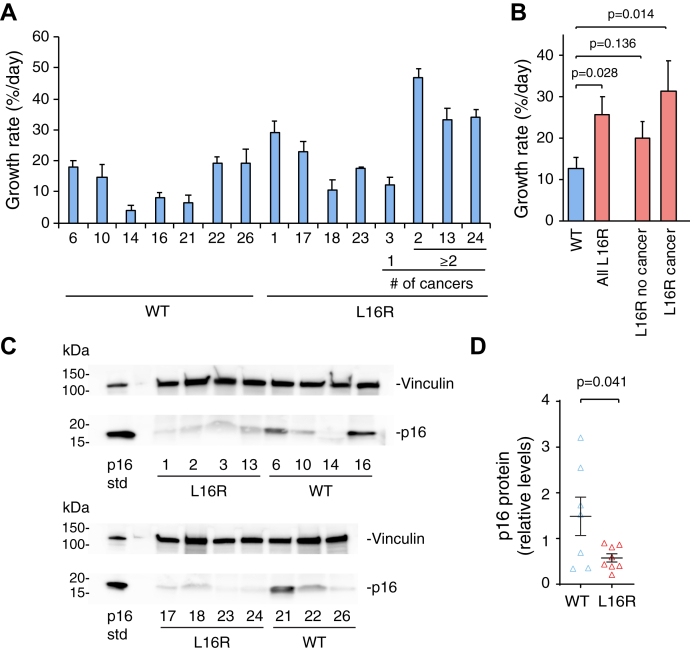
**Fibroblasts from p16-L16R carriers exhibit faster growth and express less p16 protein than WT fibroblasts.** Human skin fibroblasts (HSFs) were isolated from dermal biopsies collected from p16-L16R and WT donors in p16-L16R families. Fibroblasts from eight p16-L16R and seven p16-WT individuals were cultured through multiple passages. *A*, proliferation rates of individual HSFs. HSFs were plated at equal numbers and cultured for 1 week, then trypsinized, counted, and then replated at equal numbers. Results were expressed as percent growth (increase in cell number)/day from passages 12 to 15. Mean ± SE over these weeks are shown for each individual HSF culture. The numbers below each bar indicate codes for the different individually derived HSFs. Four HSFs from L16R donors who had occurrence of cancer are shown at *right* and the number of cancers (# of cancers) experienced by each individual is indicated. All other HSF donors had no documented cancers. *B*, average values (mean ± SE) for p16-WT and all p16-L16R groups calculated from values in *A*. Values for p16-L16R HSF subsets with no cancer (n = 4) and with cancer occurrence (n = 4) are also shown separately at *right*. *C*, lysates were prepared from HSFs cultured 14 days without media change and western blotted for p16. Equal protein was loaded per lane. Vinculin was blotted as a housekeeping protein. At *left* (p16 std), a sample containing untagged p16 overexpressed in Panc1 cells was loaded as a positive p16 standard. *D*, quantitation of p16 levels from the blots shown in *C*. Results shown are individual replicates (*triangle markers*) and means ± SE (*black crossbars*) and are relative values normalized to equal amounts of p16 std loaded on each blot. *Brackets* with *p* values indicate groups that were compared in two-tailed *t*-tests.

To evaluate the impact of p16-L16R expression on growth, passage 12 fibroblasts from individual donors were plated at equal numbers (6 × 10^5^ cells/10 cm dish), cultured for a week, trypsinized, counted, and then replated as above. This process was repeated until HSFs stopped replicating. Average growth rates from passages 12 to 15 were significantly higher for p16-L16R than p16-WT cells, (25.8 *versus* 12.8% increase in cell number per day, respectively) (Fig. 4, *A* and *B*). Growth rates of individually derived fibroblasts from weeks 12 to 30 are shown in Fig. S5*A*. Notably, several L16R fibroblasts from patients who had been diagnosed with two or more cancers (HSFs 2, 13, and 24) exhibited the highest growth rates (Fig. 4*A*, Fig. S5*A*). When averaged separately, the four HSFs from individuals who had one or more cancers (all L16R) had significantly higher growth rates (31.5%/day) than WT HSFs (Fig. 4*B*). Growth rates of p16-L16R HSFs from donors with no cancer (20%/day) were higher than average WT values, but not significantly so. Beginning at passage 16, some fibroblasts ceased to proliferate. However, a trend toward higher growth rates for p16-L16R samples *versus* p16-WT fibroblasts could be seen at almost all passages (Fig. S5*A*). We also evaluated cell proliferation rates of selected WT and L16R HSFs by FACS analysis. Individual WT HSFs exhibited a smaller percentage of cells in G2 and S phases than L16R fibroblasts (Fig. S5*B*). When pooled, L16R HSFs were significantly higher in percent S phase and G2 + S phases than WT HSFs (Fig. S5*C*), supporting our findings of faster cell proliferation rates in p16 L16R *versus* WT HSFs as measured by cell counting (Fig. 4, *A* and *B*).

By passage 30, 5/7 p16-WT cells had ceased to proliferate, but only 2/8 p16-L16R cells had stopped growing (Fig. S5*A*). The average terminal passage was 29.9 from among p16-L16R carrier fibroblasts and 25 for p16 WT HSFs (Fig. S6*A*). Together, these results indicate that on average p16-L16R-expressing fibroblasts had greater growth rates and tended to survive to later passages than p16-WT cells. Although samples sizes are small, the data suggest that p16-L16R expression confers a greater proliferative capacity on cells. Proliferation rates of individual fibroblasts were inversely correlated with patient age (r = −0.59. *p* = 0.04) when samples with multiple cancers and the greatest growth rates were excluded (Fig. S6*B*), consistent with a relationship between fibroblast growth rate and donor age reported in previous studies (29, 30). However, when the three outlying HSF samples from patients with multiple cancers were included in this analysis, no significant correlation between age and growth rate (r = −0.03, *p* = 0.04) was observed (Fig. S6*B*).

We also investigated the impact of carrying the p16-L16R allele on p16 protein expression in HSFs. At low passages, little endogenous p16 protein was detectable by western blotting in WT or L16R fibroblasts (Fig. S7*A*). By passage 7, p16 was detected in some samples of both genotypes. In HSFs (passage 11) cultured for 1 week in normal media, p16 protein levels were decreased in L16R *versus* WT-p16 fibroblasts, but differences between groups were not statistically significant (Fig. S7*B*). Challenging cells with nutrient depletion is reported to increase p16 protein expression in fibroblasts (31, 32). Thus, fibroblasts were cultured for 14 days with no changes to fresh medium. Under this condition, p16-L16R samples displayed significantly lower p16 protein expression than p16-WT fibroblasts (Fig. 4, *C* and *D*). Thus, the occurrence of heterozygous p16-L16R in HSFs can lead to an overall decrease in endogenous p16 protein expression, consistent with our findings using ectopically overexpressed p16 WT and L16R in multiple cell types. Since we had observed that the proteasome inhibitor, bortezomib, protected overexpressed p16-L16R from degradation (Fig. S3, *C* and *D*), we treated HSFs endogenously expressing WT or heterozygous L16R p16 with bortezomib and evaluated the effects by western blotting. However, bortezomib had no impact on endogenous protein levels of WT or L16R p16 in HSFs (Fig. S8*A*) and also had no impact on p16 protein levels of p16-WT (501MEL and HEK293) or p16-L16R (RPMI-7951) cells (Fig. S8*B*). Thus, the decreased levels of endogenous p16 detected in p16-L16R heterozygous HSFs do not appear to be due to bortezomib-sensitive proteasomal degradation.

## Discussion

We identified the *CDKN2A*(47T>G)/p16-L16R variant in four multigeneration kindreds of Mayo Clinic patients that contained members diagnosed with familial pancreatic cancer and melanoma. Here, we demonstrated that when expressed in p16-null pancreatic cancer cells, p16-L16R had little ability to suppress the cell cycle and exhibited decreased binding to CDK4 and CDK6 compared with p16-WT. These findings provide evidence that p16-L16R is a *bona fide* deleterious mutation, consistent with the association of this variant with increased occurrence of melanoma and pancreatic cancer. In addition, we found that p16-L16R exhibited lower protein levels compared with the WT protein, as demonstrated by ectopic expression in cells. HSFs with heterozygous p16-L16R also showed higher proliferation and lower overall levels of p16 protein, consistent with the findings in pancreatic cancer cells. Thus, through its inability to suppress the cell cycle and decreased protein expression, the presence of p16-L16R leaves carriers at higher risk of melanoma and pancreatic cancer.

We showed that p16-L16R protein was consistently detected at a lower level by western blotting compared with p16-WT, when expressed in pancreatic cancer cells and a melanoma cell line. mRNA levels of overexpressed WT and L16R p16 were not significantly different when plasmids were transfected at equal DNA levels, suggesting that decreased protein expression of p16-L16R is regulated at the posttranslational level. In addition, p16-L16R heterozygous fibroblasts also expressed lower levels of total p16 than did p16-WT HSFs when cells were grown under nutrient-limiting conditions. Our *in silico* molecular modeling studies showed that p16-L16R is likely less stable than p16-WT. Although p16-L16R protein was protected from degradation by the proteasomal inhibitor, bortezomib, when overexpressed in cell lines, bortezomib had no protective effect on endogenously expressed p16 WT or L16R. Thus, the lower levels of endogenous p16-L16R in HSFs do not appear to involve rapid proteasomal degradation. We also demonstrated that p16-WT is not protected from degradation by binding to CDK4/6, ruling out the possibility that p16-L16R is degraded more quickly because it is not bound to CKD4 and CDK6. Interestingly, we note that several other p16 variants of unknown significance identified as associated with pancreatic cancer (2, 25) also exhibited lower protein levels, compared with p16-WT (Fig. S4). The mechanisms that control the decreased protein expression of p16-L16R and other variants and the consequences of lower expression for carriers of these variants will require further study.

To our knowledge, our studies are the first to investigate the impact of endogenous germline p16 mutants on cell proliferation using fibroblasts from normal skin biopsies of individuals in familial melanoma/pancreatic cancer families. Earlier studies of fibroblasts from cancer patients of unknown germline mutation status and investigations of p53 mutant HSFs have shown that cells from cancer patients possess altered growth characteristics compared with normal fibroblasts (33, 34). One study previously investigated transcriptomic alterations in p16 mutant fibroblasts compared with unrelated WT controls, but did not report growth characteristics (35). Our utilization of fibroblasts from p16-L16R and p16-WT members of p16-L16R kindreds provided a unique opportunity to evaluate the impact of p16-L16R as expressed endogenously on cell proliferation and p16 protein expression. Proliferation assays demonstrated that p16-L16R HSFs on average grew faster than WT HSFs, whereas western blotting showed that p16-L16R fibroblasts on average expressed lower p16 protein than did WT cells. These studies suggest that decreased function and lower protein expression of p16-L16R HSFs lead to a greater proliferative capacity than seen in WT HSFs. We note that the p16-L16R HSFs with the highest growth rates were those from individuals who had a history of multiple cancers. Because of our small sample size, we cannot discount the possibility that HSFs from individuals with past cancers were affected systemically, by either exposure to cancer or treatments for the malignancies (*e.g.*, chemotherapy). p16-L16R patients without cancer (20% growth per day) showed a trend (*p* = 0.13 in two-tailed *t*-tests) toward higher growth rates than WT cells, supporting the idea that p16-L16R increases growth rates, even in subjects without cancer. Additional studies using larger samples sizes are needed to distinguish between direct p16-L16R effects on carrier HSFs and systemic effects due to previous cancers.

Further research is needed to understand why certain individuals carrying p16-L16R or other germline *CDKN2A* mutations develop melanoma, pancreatic cancer, or other malignancies and why penetrance of these mutations is less than 100% (2, 36, 37, 38). Proposed factors involved in variations in penetrance and presentation include genetic factors such as other mutations or gene variants, environmental factors such as exposure to UV, smoking, or toxic chemicals. Skin fibroblasts derived from p16 WT and mutant members of p16 mutant familial melanoma/pancreatic families could be a useful model for studying such factors. For example, exome sequencing or transcriptomic studies might be ideally performed using first-degree relatives that are p16 WT *versus* mutant to minimize genetic variation between individuals.

In conclusion, we employed ectopic expression of p16 WT and L16R in pancreatic cancer cells, and normal fibroblasts derived from individuals in families that segregate p16-L16R to provide molecular evidence that p16-L16R is a deleterious mutation with little ability to interact with CDK4/6 or suppress cell cycle progression. This mutation is likely to act as a “first hit” promoting the eventual biallelic loss of p16 function that frequently occurs in pancreatic cancer and melanoma. It is also possible that expression of p16-L16R leaves cells more susceptible to malignant transformation due to faster proliferation rates or other losses in p16 tumor suppressor function.

## Experimental procedures

### Patients and patient samples

All aspects of the study involving human subjects were reviewed and approved by the Mayo Clinic Institutional Review Board (IRB). Written informed consent was obtained from all participants. All participants provided a lymphocyte DNA sample, which was tested for the presence/absence of the p16-L16R variant by Sanger DNA sequencing and confirmed by commercial genetic testing (16, 17). Subjects completed a demographic and risk factor questionnaire. The resulting data were anonymized and abstracted to ascertain history of cancer and age of participants.

Skin biopsies were taken from volunteers from among the kindred families, including those with WT or L16R p16 status and those with or without a history of cancer. Skin punch biopsies (4 mm) were performed on a sun-unexposed area of the upper arm using sterile technique and a topical anesthetic/lidocaine and/or injectable methyl paraben-free lidocaine (placed in the skin using a needle) to minimize patient discomfort, followed by closing with 1 to 2 sutures. Samples of punch biopsies were placed in a sterile 15 ml conical test tube with biopsy transport media [Roswell Park Memorial Institute Medium (RPMI) with 1% antibiotic-antimycotic]. Sample tubes were labeled and sealed with Parafilm and stored at 4 °C for up to 3 days until processing.

Biopsy dissection and initial fibroblast culture were conducted under sterile conditions by the Mayo Clinic Biochemical Genetics Laboratory. Skin samples were dissected into 12 to 15 small pieces using sterile scalpel and forceps. Pieces of sample were transferred to a cell culture flask and incubated at 37 °C in a CO_2_ incubator for several days in growth media (EMEM, 20% FBS, penicillin, streptomycin, amphotericin B, gentamicin). After 1 week, cells were washed in PBS and passed using trypsin. Cell culture in growth media was continued until all cultured cells were fibroblasts (keratinocyte growth is inhibited under these conditions). After 2 to 3 passages, samples of HSFs were cryopreserved for reference or later use. Actively growing HSFs were used for cell biological, biochemical, and molecular studies. To verify the p16 status as WT *versus* L16R for each HSF, genomic DNA was isolated from HSFs using the DNeasy Blood & Tissue Kit (QIAGEN cat: 69506). PCR was then conducted using the Terra PCR Direct Red Dy Premix Kit (Takara cat: 639286) using the following primers: CAACCTGGGGCGACTTC (sense), CTGCAAACTTCGTCCTCCAG (antisense). PCR products were run on 1% Agarose gels, and ∼483 bp fragments containing a single band were then extracted using the Gel/PCR DNA Fragment Extraction Kit (IBI Scientific, catalog # IB47030). Gel extracts were submitted to GENEWIZ, and Sanger sequencing was performed using the above listed antisense primer.

### Cell lines, cell culture, and transfections

Panc1 and MiaPaca2 pancreatic adenocarcinoma cells and RPMI-7951 melanoma cells were obtained from American Type Culture Collection. HPNE cells (hTERT-immortalized human pancreatic epithelial nestin-expressing stably transfected with mutant KRAS g12V and p16 shRNA) (22) were a gift from Paul Chiao, MD Anderson Cancer Center. All cell culture media were from Corning. All cells were cultured in 5% CO_2_ at 37 °C. Panc1 were cultured in DMEM with 10% heat-inactivated fetal bovine serum (FBS, SAFC BioScience), RPMI-7951 cells in EMEM/10% FBS, and HSFs in DMEM/10% FBS plus 1:100 Penicillin-Streptomycin (Thermo). Cell lines in normal growth media (8 × 10^5^ in 10 cm culture dishes) were transiently transfected utilizing XtremeGene HP (MilliporeSigma) using a ratio of 1 μg plasmid DNA/3 μl XtremeGene HP/ml of growth medium. CDK4 and CDK6 depletion was performed using Dharmafect1 reagent (Dharmacon) according to manufacturer's instructions with siCDK4 and siCDK6 ONTARGETplus SMARTpools, siRNAs (Dharmacon), and Allstar Neg. control siRNA (catalog # 1027281, Qiagen).

### Plasmid and mutant constructs

Plasmid vector pQCXIH (Addgene plasmid 37104) containing WT p16 was purchased from Addgene and cloned into p3XFLAG-CMV-14 (Sigma-Aldrich) using the following primers and restriction sites: (EcoRI) ATAAGAGAATTCATGGAGCCGGCGGCGGGGAGC (sense) and (XbaI), CTAGCTTCTAGAATCGGGGATGTCTGAGGGACCTTCCGCG (antisense), and into the pIRES2-EGFP bicistronic vector (Clontech) using (Not1) ATAAGAATGCGGCCGCATGGAGCCGGCGGCGGGGAGCAG (sense) and (EcoR1) ATAAGAGAATTCTCAATCGGGGATGTCTGAGGGACCTTCCGCG (antisense). Mutant L16R was prepared using the Q5 Site-Directed Mutagenesis Kit from New England Biolabs. The following primers were used: sense GCTGACTGGCGGGCCACGGCC and antisense CGAAGGCTCCATGCTGCTCCC. Other p16 variants were prepared similarly.

### Western blotting

All cells were lysed in lysis buffer [50 mM Tris, pH 7.5, 1% NP-40, 0.1% SDS, 300 mM NaCl, 2.5% glycerol, 1 mM DTT, 1 mM MgCl_2_, supplemented with 10 μg/ml leupeptin, 10 μg/ml aprotinin, 1 μg/ml pepstatin, 1 mM phenylmethyl sulphonyl fluoride, and complete protease inhibitor cocktail (unless otherwise noted all reagents are from MilliporeSigma)]. For HSFs, 1 to 2 confluent 10 cm dishes were washed three times with ice-cold PBS. Cells were then were scraped in 80 μl lysis buffer. For transfected cells, 2 days after transfection, cells were washed three times with ice-cold PBS and then lysed in 150 μl lysis buffer. Protein lysates were dispersed by passage three times through a 25-gauge needle, then centrifuged at 17,000*g*, and the supernatant collected. Protein concentrations of cleared lysates were determined using the BCA protein reagent (Pierce). Lysates were then diluted in Laemmli buffer with 20 mg/ml DTT and run on 12% or 4 to 20% Bio-Rad Min-Protean TGX or Criterion TGX unstained precast gels. Gels were transferred to PVDF membranes in Towbin transfer buffer with 20% MeOH. The transferred membranes were blocked in 3% BSA in PBS with 0.3% Tween-20 (PBS-T) for 1 h. Membranes were then incubated at room temperature for 2 h with primary antibodies in PBS-T with 3% BSA. The following primary antibodies were used: M2-FLAG (catalog # F1804), vinculin (catalog # 2669856) and α-tubulin (catalog # T6199) (all from MilliporeSigma), and mouse anti-p16 (BD Biosciences, San Jose, CA; catalog # 554079). After washing, blots were incubated for 1 h with horseradish peroxidase secondary antibodies (MilliporeSigma) in PBS-T with 3% BSA. After further washes, immunoreactive signals on blots were detected with Thermo SuperSignal West Pico and West Dura Chemiluminescent Substrate kits. Signals were captured on X-Ray film and converted to digital images by scanning or scanned using a Bio-Rad ChemiDoc MP system. Blot signals were quantified using Image J 1.49v.

### Immunoprecipitation

Panc1 cells transfected for 2 days with WT or L16R p16 were lysed in lysis buffer containing 300 mM NaCl, then diluted with lysis buffer without NaCl to a final concentration of 150 mM NaCl. Lysates were then precleared by incubation for 1 h with 20 μl of Dynabeads-Protein G (Thermo) at 4 °C. M2-Flag antibody or nonimmune mouse IgG (2 μg per tube) was prebound to 30 μl of Dynabeads-Protein G in 400 μl PBS/0.05% Triton X-100 for 1 h at room temperature, followed by washing once in 400 μl of PBS/0.05% Triton X-100. Dynabeads–antibody complexes and lysates were combined and incubated overnight at 4 °C with rotation. Bead complexes were then washed three times in 25 mM sodium citrate, 50 mM dibasic sodium phosphate, pH 5.0. Protein complexes on beads were eluted by addition of SDS sample buffer and incubation at 95 °C for 5 min. These eluates were subjected to SDS-PAGE and western blot analysis as above using M2-Flag and antibodies against CDK4 (Upstate Biotechnology, Lake Placid NY; catalog # 06-139) and CDK6 (Cell Signaling Technology, Danvers MA; catalog #13331S).

### Immunofluorescence

PANC1 cells cultured on 25 mm coverslips in 35 mm culture dishes were transfected for 48 h with WT or L16R p16 in the pCMV-FLAG vector. Cells were then fixed in 3.2% formaldehyde for 40 min and permeabilized in 0.1% Triton-X-100 at 4 °C for 5 min. Subsequent steps were performed at room temperature. Samples were blocked for 1 h in blocking buffer (BB; 5% goat serum, 5% glycerol, 0.05% NaN_3_ in PBS), incubated for 2 h in M2 anti-FLAG antibody diluted 1/200 in BB, washed three times in PBS, and then incubated for 1 h with ALEXAfluor-594 goat anti-mouse secondary antibody (Thermo) at 1:200 in BB. After staining, these were washed three times in PBC, and then samples were mounted on microscope slides in Prolong with DAPI (Thermo). Microscopy was performed using a Zeiss system including an Axiovert 200 microscope with a 20× Plan-APO Chromat lens and an Axiocam 702 mono camera. Images were captured using the Zen 2.3 application. Image fields were taken randomly in red (AF546; immunofluorescence) and blue (DAPI) channels, using only the blue signal for focusing. For each coverslip, 14 fields were acquired. DAPI and immunofluorescent images were overlaid and quantified using Adobe Photoshop CC 19.1.4. A minimum of 790 total cells were counted per coverslip. Results were expressed as percent transfection [100 × number of red (p16-positive) cells/total blue nuclei].

### Cell cycle analysis

Panc1, MiaPaca2, and HPNE cells were transfected as above with p16-FLAG (WT or L16R) or pCMV vector only, plus EGFP-N1 at a ratio of 8:1 (p16:EGFP). After 1 day, cells were trypsinized and replated at 2 × 10^5^ cells/well in 6-well plates. For Panc1 and MiaPaca2 cells, 2 days after transfection, cells were washed with cold PBS, trypsinized, and centrifuged at 500*g* for 5 min. Cell pellets were fixed by resuspending in 1 ml of PBS with 2% formaldehyde, 2% glucose for 15 min on ice. The cells were washed with cold PBS and centrifuged at 500*g* for 5 min, followed by resuspension in 500 μl of PBS with 0.1% sodium citrate, 0.1 mg/ml RNAse, 0.05% Triton, and 50 μg/ml propidium iodide for 1 h at 4 °C in the dark. For HPNE cells and HSFs (not transfected), 2 day after transfection, cells were washed with cold PBS, trypsinized, and centrifuged at 1500*g* for 5 min. Cell pellets were fixed by adding 430 μl of cold PBS and then adding 1 ml of 100% EtOH, dropwise. The cells were then kept at −20 °C overnight. The next day, cells were washed with cold PBS and centrifuged at 1500*g* for 5 min, followed by resuspension in 300 μl of PBS with 0.9 ng/μl RNAse and 0.5 ng/μl propidium iodide. FACS was performed using BD FACSCanto II Flow Cytometry System, using BDX Diva software. For each measurement, 40,000 (Panc1 and MiaPaca2) or 20,000 (HPNE) events were acquired. Analysis of cell cycle was performed using the Modfit cell cycle program (Verity Software House) to determine the fractions of cells in the G1, S, and G2/M phases from the cell cycle distribution.

### Proliferation studies

Passage 12 fibroblasts from individual donors expressing either WT or heterozygous p16-L16R were trypsinized, counted using a manual hemocytometer, centrifuged, and resuspended in fresh DMEM/10% FBS with 1:100 pen-strep. Cells were plated in fresh media at equal numbers (6 × 10^5^ cells/10 cm dish) and cultured for 1 week. Cells were trypsinized, counted, and replated as above once per week at 6 × 10^5^ cells/dish until they ceased proliferating. Proliferation rate (% increase in cell number per day) was calculated by the formula: 100 × (number of cells recovered/the number of cells plated the week before)/7 days per week.

### Quantitative reverse transcription–PCR (q-PCR)

Total RNA was extracted from Panc1 cells using TRIzol reagent (Invitrogen). The High-Capacity cDNA Reverse Transcription kit (Applied Biosystems) was used to reverse transcribe 2 μg of RNA. A portion of the total cDNA was amplified by real-time PCR. Samples were prepared with PerfeCTa SYBR Green FastMix (Quanta BioSciences Inc) and the following primers were used to detect p16-FLAG: AATGTCGTAATAACCCCGCCCCGTTGACGC (sense); CGAAGGCTCCATGCTGCTCCC (antisense); and the housekeeping genes TBP: GGTTTGCTGCGGTAATCATGA (sense), CTCCTGTGCACACCATTTTCC (antisense); HPRT: TGGAAAAGCAAAATACAAAGCCTAAGATGA (sense), ATCCGCCCAAAGGGAACTGATAGTC (antisense). Amplification was performed using the C1000 Thermal Cycler (Bio-Rad) with BioRad CFX Manager software. p16 RNA levels were calculated following the 2ΔCt method using the corresponding levels for HPRT and TBP in the same sample.

### Molecular dynamics simulations

For the MD simulations, we used the NMR solution structure of human p16 (PDB code 2A5E) (19) as starting model. The N-terminal (residues 1–7) and C-terminal (residues 140–156) disordered regions of the protein were omitted from the MD simulations. Coot was used to generate the p16 L16R mutant (39). The MD simulations were performed and analyzed with GROMACS (version 5.1.2) (40) using the all-atom CHARMM27 force field (41). The aqueous environment was simulated with explicit TIP3P water molecules in triclinic boxes (5.27 × 6.42 × 6.13 nm^3^) with a solute-wall minimum distance of 1.0 nm. Charges were neutralized with Na^+^ and Cl^−^ atoms with 100 mM NaCl included to approach physiological conditions. There were 20,808 atoms in the WT system and 20,809 atoms for the simulation of L16R.

The systems were subjected to steepest decent energy minimization with a maximum force of 200 kJ/mol/nm. The temperature and volume of each system were then equilibrated by running 200 ps of constant volume and constant temperature (NVT) equilibration at 298 K with a velocity-rescaling thermostat. This was followed by equilibration for 1 ns to a 1.0 bar constant pressure (NPT) bath using the Berendsen weak coupling method (42). The above equilibration steps were position-restrained on protein molecules. The MD simulations used periodic boundary conditions with a time step of 2.0 fs. The cutoff for nonbonded interactions was 1.0 nm. Long-range electrostatic interactions were calculated using the particle mesh Ewald (PME) method (43) with a Fourier grid spacing of 0.12 nm. The LINCS algorithm (44) was used to control the bond lengths, and a leap-frog integrator was used for all the simulations. Trajectories were written every 20 ps.

### Statistical analysis

All data calculations and statistical analyses were performed using Microsoft Excel version 16.16. 26. Results with multiple replicates were expressed as mean ± standard error (SE).

## Data availability

All the data described are located within this article.

## Supporting information

This article contains supporting information.

## Conflict of interest

The authors declare that they have no competing interests with the contents of this article.
