# Supplementary material for: A rare germline CDKN2A variant (47T>G; p16-L16R) predisposes carriers to pancreatic cancer by reducing cell cycle inhibition
Source: J Biol Chem. 2021 Apr 3;296:100634. doi: 10.1016/j.jbc.2021.100634 (PMC8121974; doi:10.1016/j.jbc.2021.100634)
Supplement: Supplemental Figures S1–S8 [file mmc1.pdf]

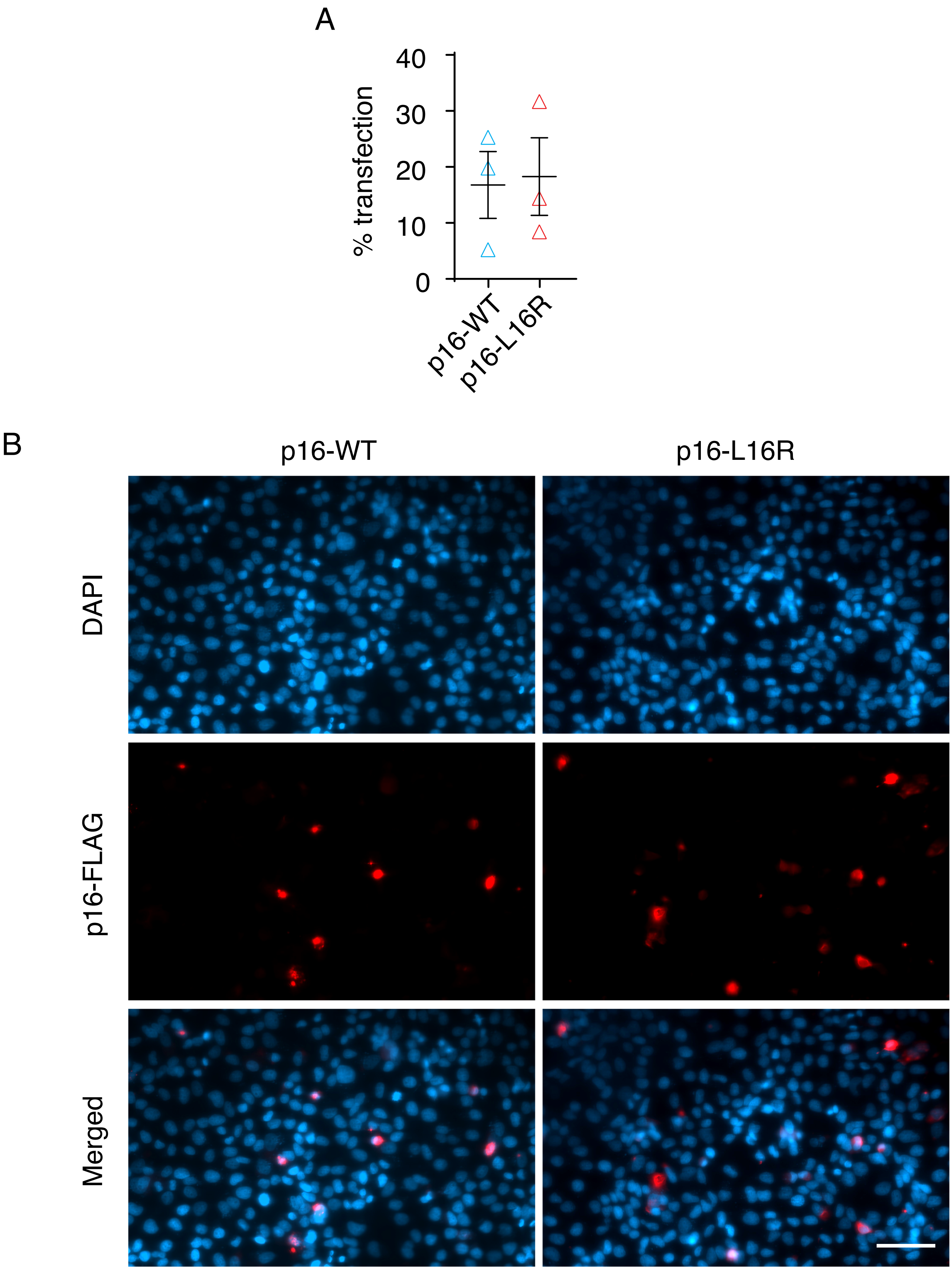

Supporting Figure 1. Estimation of p16-FLAG transfection efficiency by immunofluorescence. Panc1 cells were transfected for 48 h with FLAG-tagged WT or L16R p16 and then processed for immunofluorescence using an antibody against the FLAG epitope. A) Fields of cells on cover slips were photographed randomly under a fluorescence microscope using only the DAPI signal for focusing. 3 independent experiments were performed. In each experiment, at least 14 fields each of WT and L16R transfected cells were photographed in the blue (DAPI) and red (p16) channel. Blue and red images were merged in Adobe Photoshop and the number of total (DAPI nuclei) and p16-positive cells counted. At least 790 total cells were counted per replicate for each group. Results shown are values from individual experiments (triangle markers) and mean±SE percent transfection. B) Representative images. bar=50 μm.

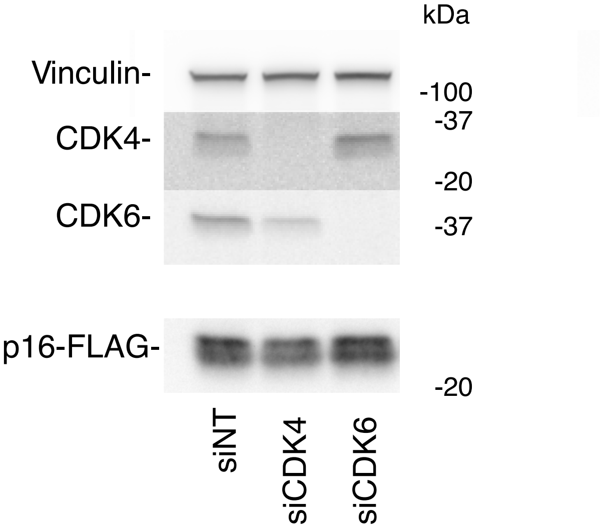

Supporting Figure 2. p16-WT is not destabilized by loss of CDK4 or CDK6. Panc1 cells were treated with CDK4, CDK6 or non-targeting control (NT) siRNA (total 3 days) and transfected with p16-WT-FLAG (total 2 days). Cells were lysed and analyzed by western blotting for CDK4, CDK6 and p16-FLAG. Vinculin is shown as a protein loading control. The knockdown of CDK4 or CDK6 did not cause a significant decrease in p16-WT levels.

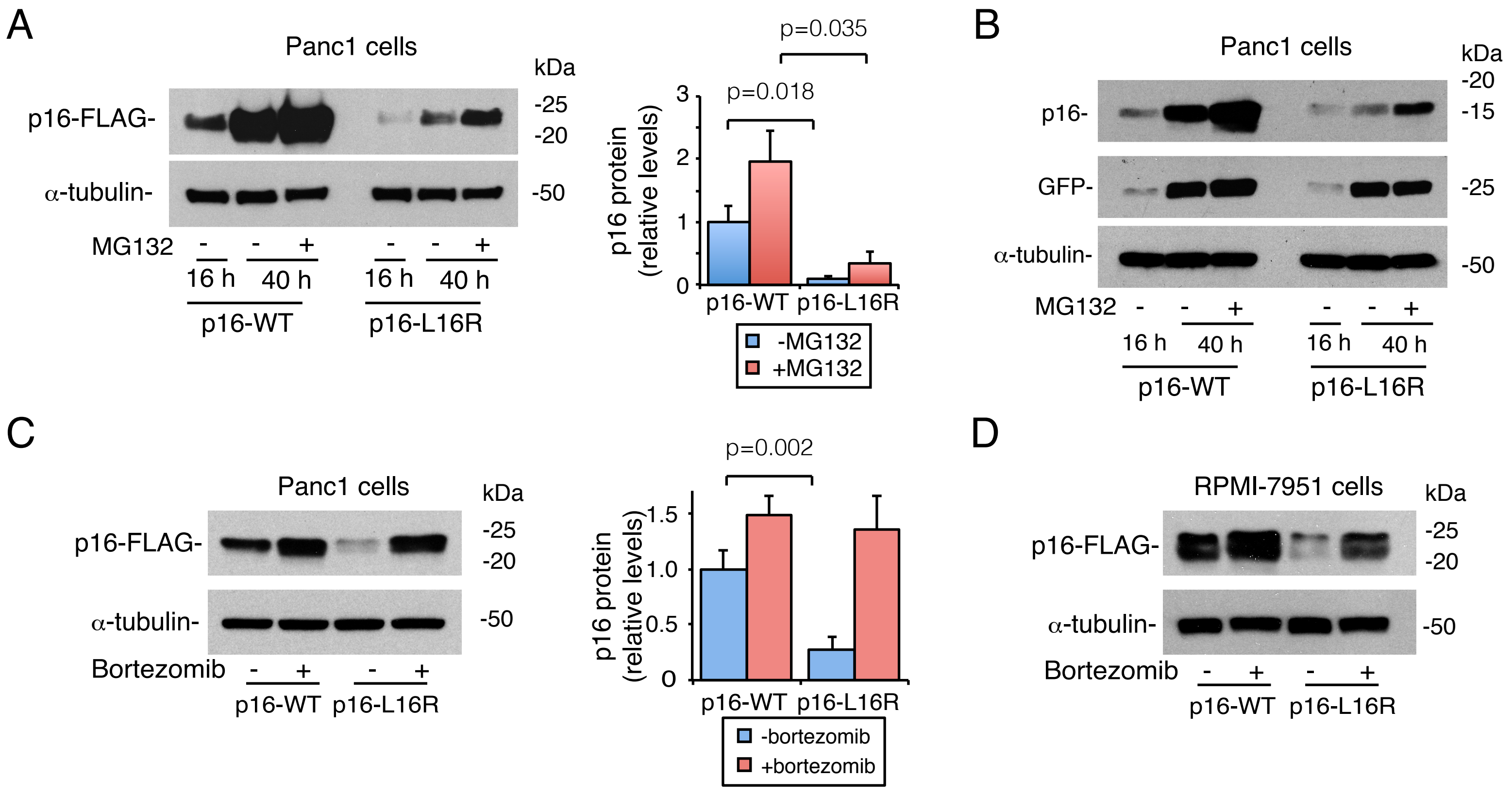

Supporting Fig. 3. Effects of proteasomal inhibitors on p16 WT and L16R protein stability. (A) Panc1 cells were transfected with equal plasmid DNA of WT or L16R p16-FLAG and were either lysed at 16h, or after 24 h were treated with 10 μM MG132 or equivalent DMSO for an additional 16 h before lysis (40 h samples). Cell lysates were then analyzed by western blotting. Left panel: western blot showing that MG132 increases protein levels of both L16R and WT p16 but does not stabilize L16-p16 up to WT levels. Right panel: quantitaition of p16 protein expression at 40 h from western blots. N=3 for each condition. Results expressed as mean ± SE normalized to the value for WT expression. (B) Cells were transfected with bicistronic pIRES2-GFP vector encoding untagged p16 (WT or L16R) plus GFP. Cells were treated as in A and then western blotted, showing that untagged L16R-p16 exhibited lower expression than WT-p16 when detected using a p16 antibody, and that both p16 proteins showed some stabilization with MG132. GFP showed similar levels whether it was coexpressed with WT or L16R p16 proteins. (C). Panc1 cells were transfected with FLAG-tagged WT and L16R p16 at equal μg of plasmid DNA. After 24 h transfection, cells were treated with 100 nM bortezomib, or equivalent DMSO vehicle for 16 h. Cell lysates were then prepared and western blotted for FLAG-p16. Left panel: western blot showing that bortezomib treatment restored p16-L16R to a level similar to that of the WT. right panel: Quantitation of western blot signals for p16. N=5 for each condition. Values are mean ± SE expressed relative to the mean value of p16-WT treated without bortezomib. D) RPMI-7951 melanoma cells were transfected with p16 variants and treated ± bortezomib as in C.

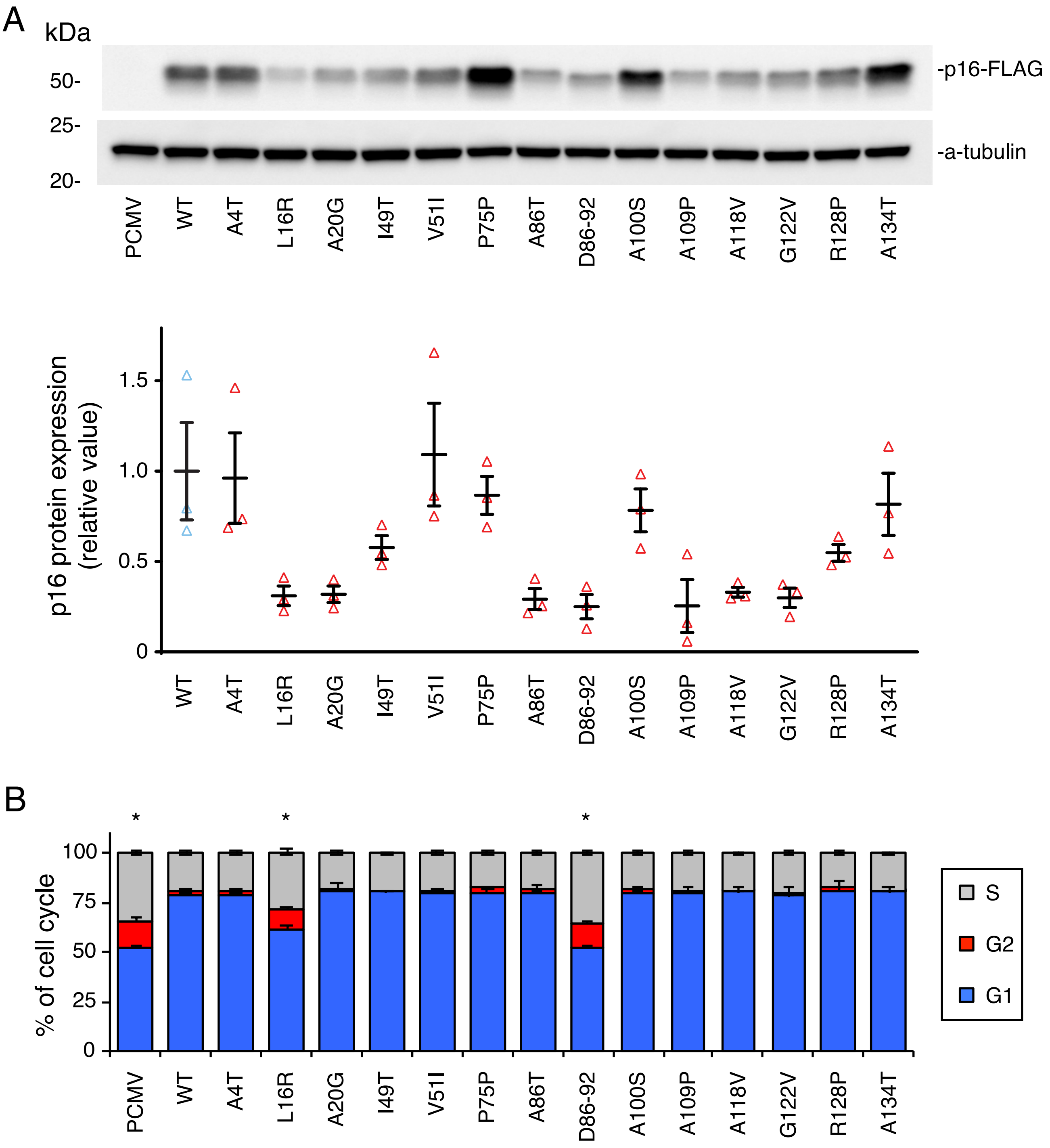

Supporting Figure 4. Expression and cell cycle suppression by p16 variant proteins. PANC1 cells were transfected for 40 h with constructs encoding FLAG-tagged p16 WT and variants. (A) Western blotting of p16 WT and variants. Upper panel: representative western blots. Molecular weight marker positions are shown at left. Lower panel shows quantitation of p16 signals on western blots. Results were calculated as relative intensity compared to all p16 signals on a single blot. Values are mean ± SE. n=3 for each group. (B) PANC1 cells were cotransfected with p16 variants or pCMV vector control, plus GFP to identify transfected cells. 40 h after transfection, cell cycle analysis was performed using FACS. Cell cycle results are means ± SE of ≥ 3 experiments for each condition. * indicates groups that were significantly different ($p<0.001$) in G2 values from WT samples in 2-tailed t-tests.

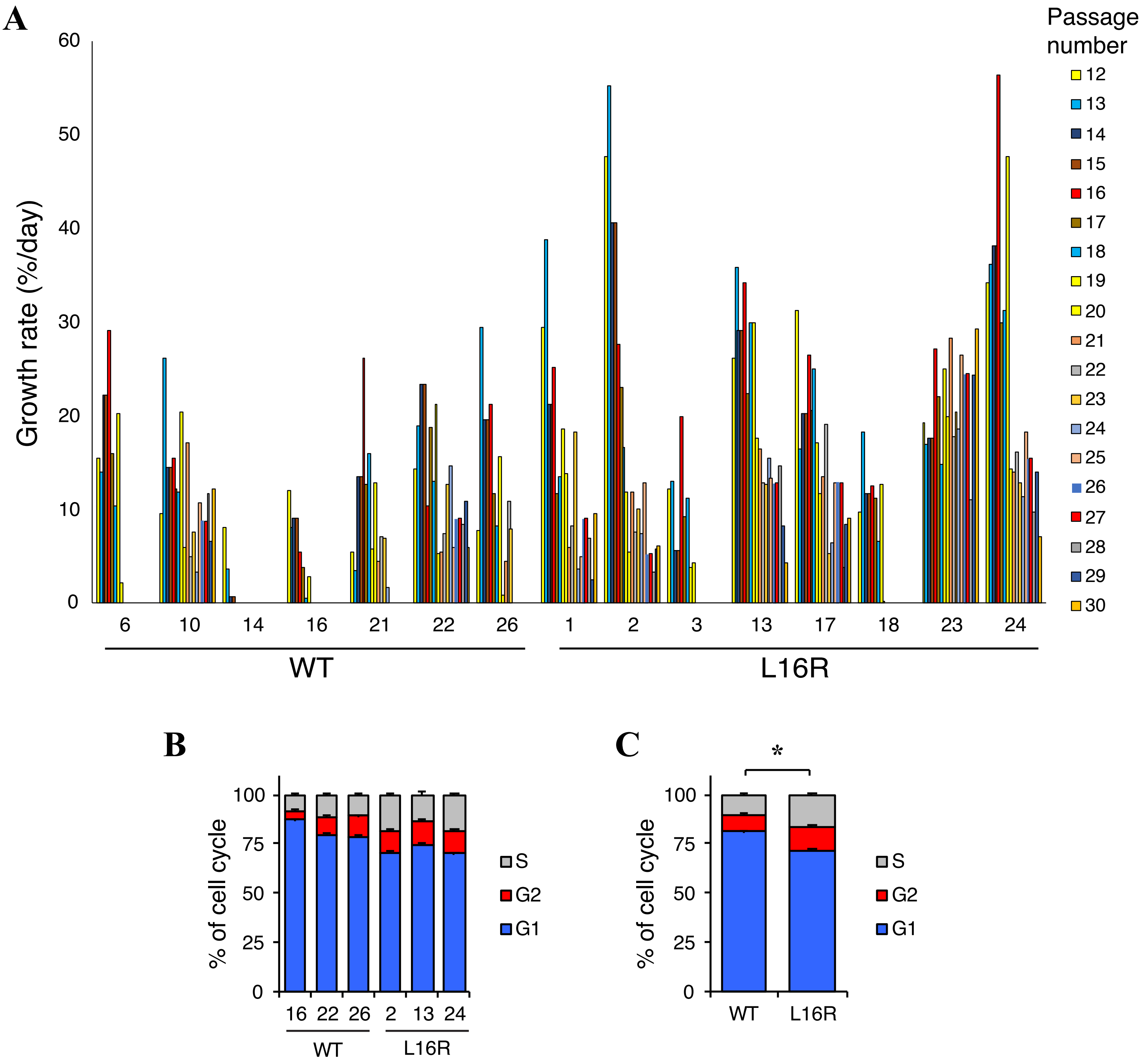

Supporting Figure 5. Growth and proliferation rates of individual WT and p16-L16R. (A) Human skin fibroblasts (HSFs) from dermal biopsies collected from p16-L16R and WT donors were cultured through multiple passages. HSFs were plated at equal numbers and cultured for 1 week, then trypsinized, counted, then replated at equal numbers, with this process repeated each week. Results are expressed as percent growth/day for passages 12-30. The numbers below each bar indicate codes for the different individually-derived HSFs. Gaps indicate HSFs that ceased replicating. (B) Cell cycle analysis of selected WT and L16R HSFs **(passages 13-19)** was performed using FACS. Results show mean ± SE for individual HSFs noted by their number codes beneath bars, and are expressed as percentages of the population in each cell cycle phase. n=4 for each HSF. C) Mean ± SE values are shown for the data in B grouped by WT and L16R status (n=3 each). * indicates that WT and L16R HSFs were significantly different in G1 (p=0.029), S (p=0.027) and G2+S (p-0.029) in 2-tailed t tests.

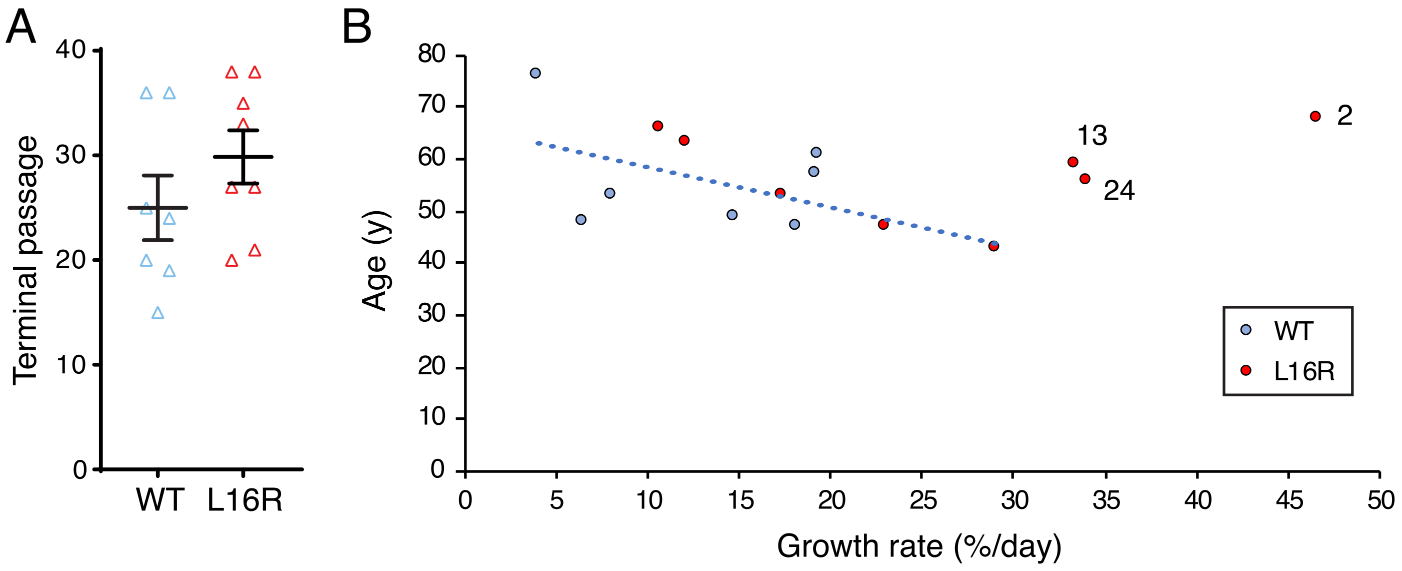

Supporting Figure 6. Terminal passage and relation between growth rate and age for individual WT and p16-L16R fibroblasts. Human skin fibroblasts (HSFs) were cultured as described in Figure 4 and Supporting Figure 3. (A) Each individual fibroblast was cultured until it stopped proliferating and the terminal passage recorded. Symbols are individual values for WT (n=7) and L16R (n=8) fibroblasts. Hash marks indicate mean ± SE for each group. (B) Growth rates (% increase in cell number/day) of individual fibroblasts (data from Figure 3A) plotted against age of the donor from which they originated. Among all samples (both WT and L16R), there was no significant correlation between age and growth rate (Pearson r = 0.03, p = 0.91. However, excluding the 3 samples from donors with ≥2 cancers (markers numbered 2, 13 and 24), age was significantly negatively correlated with growth rate for remaining samples (Pearson r = -0.59, p = 0.04). The dotted line shows the line of best fit of this correlation.

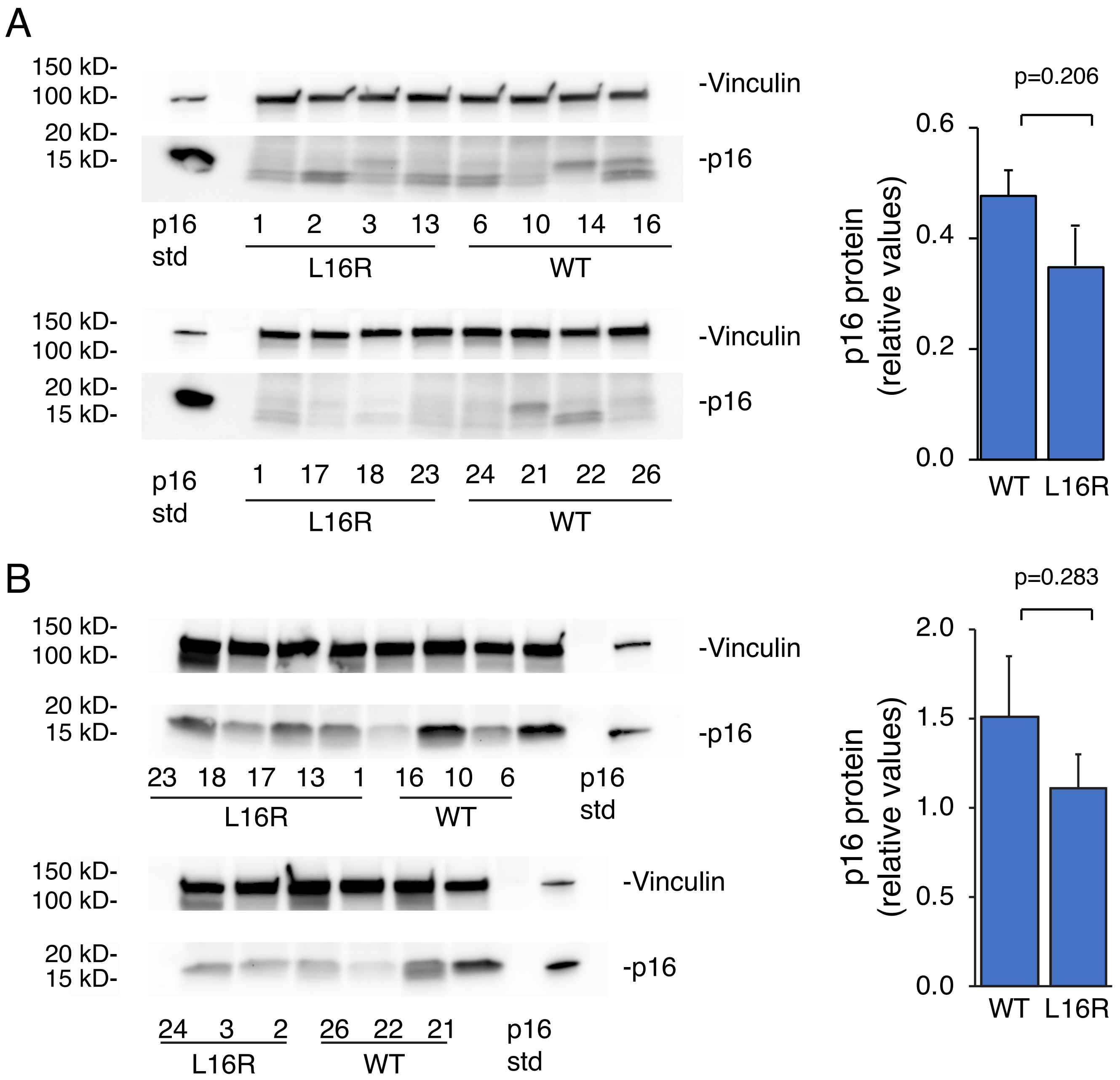

Supporting Figure 7. Endogenous p16 expression in human fibroblasts cultured for 7 days without media change. (A) Lysates were prepared from HSFs (passage 3-4) cultured 7 days with no change in media, and western blotted for p16. Equal protein (60 µg) was loaded per sample lane. (B) Lysates from HSFs (passage 11) cultured 7 days with no change in media were western blotted for p16. Equal protein (30 µg) was loaded per sample lane. Vinculin was blotted as a housekeeping protein. For A and B, a sample containing untagged WT p16 overexpressed in Panc1 cells (p16 std),was loaded as a positive p16 standard. Right panels- Quantitation of p16 levels from the blots shown at left. Results are means ± SE and are relative values normalized to equal loadings of p16 std on each blot. p values from a 2-tailed t-tests are shown.

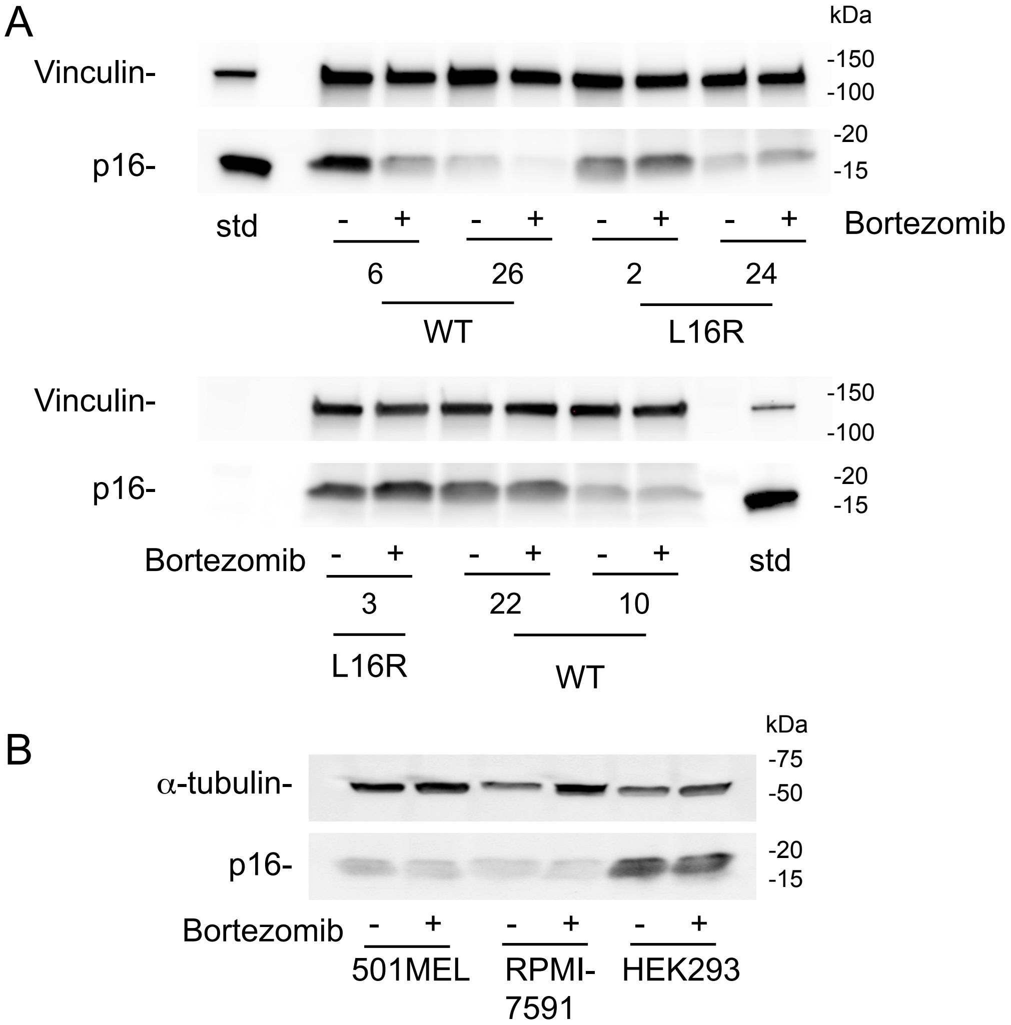

Supporting Fig. 8. Lack of effect of the proteasomal inhibitor, bortezomib, on endogenous p16 protein levels. HSFs (A) or cell lines (B) expressing endogenous p16 were treated with 100 nm bortezomib or equivalent DMSO for 24 before lysis. Cell lysates were then analyzed by western blotting for p16 protein levels. Note that RPMI-7951 cells are homozygous for p16-L16R while 501MEL and HEK293 cells express WT p16.
